# Cytokine-induced translocation of GRP78 to the plasma membrane triggers a pro-apoptotic feedback loop in pancreatic beta cells

**DOI:** 10.1038/s41419-019-1518-0

**Published:** 2019-04-05

**Authors:** Saurabh Vig, Mijke Buitinga, Dieter Rondas, Inne Crèvecoeur, Marc van Zandvoort, Etienne Waelkens, Decio L. Eizirik, Conny Gysemans, Pieter Baatsen, Chantal Mathieu, Lut Overbergh

**Affiliations:** 1 Clinical and Experimental Endocrinology, KU Leuven, Leuven, Belgium; 2 Department of Molecular Cell Biology and School for Nutrition and Translational Research in Metabolism NUTRIM, Maastricht University, Maastricht, The Netherlands; 3 Laboratory of Protein Phosphorylation and Proteomics, KU Leuven, Leuven, Belgium; 4 SyBioMa, KU Leuven, Leuven, Belgium; 5 ULB Center for Diabetes Research, Universite Libre de Bruxelles, Brussels, Belgium; 6 Electron Microscopy Platform of VIB Bio Imaging Core at KU Leuven and VIB-KU Leuven Center for Brain & Disease Research, Leuven, Belgium

## Abstract

The 78-kDa glucose-regulated protein (GRP78) is an ubiquitously expressed endoplasmic reticulum chaperone, with a central role in maintaining protein homeostasis. Recently, an alternative role for GRP78 under stress conditions has been proposed, with stress-induced extracellular secretion and translocation of GRP78 to the cell surface where it acts as a multifunctional signaling receptor. Here we demonstrate translocation of GRP78 to the surface of human EndoC-βH1 cells and primary human islets upon cytokine exposure, in analogy to observations in rodent INS-1E and MIN6 beta cell lines. We show that GRP78 is shuttled via the anterograde secretory pathway, through the Golgi complex and secretory granules, and identify the DNAJ homolog subfamily C member 3 (DNAJC3) as a GRP78-interacting protein that facilitates its membrane translocation. Evaluation of downstream signaling pathways, using N- and C-terminal anti-GRP78 blocking antibodies, demonstrates that both GRP78 signaling domains initiate pro-apoptotic signaling cascades in beta cells. Extracellular GRP78 itself is identified as a ligand for cell surface GRP78 (sGRP78), increasing caspase 3/7 activity and cell death upon binding, which is accompanied by enhanced *Chop* and *Bax* mRNA expression. These results suggest that inflammatory cytokines induce a self-destructive pro-apoptotic feedback loop through the secretion and membrane translocation of GRP78. This proapoptotic function distinguishes the role of sGRP78 in beta cells from its reported anti-apoptotic and proliferative role in cancer cells, opening the road for the use of compounds that block sGRP78 as potential beta cell-preserving therapies in type 1 diabetes.

## Introduction

Type 1 diabetes (T1D) is characterized by insulin dependence for survival due to the destruction of the insulin-producing beta cells. This destruction is immune-mediated with infiltrating leukocytes attacking the beta cells, but growing evidence suggests that the beta cell itself also plays an active role in its own destruction^1^.

We and others have demonstrated that sustained inflammation induces endoplasmic reticulum (ER) stress in beta cells, resulting in cellular dysfunction and eventually in beta cell death^1–3^. Interestingly, our group showed that cytokine-induced ER stress is paralleled by membrane translocation and secretion of the ER chaperone glucose-regulated protein 78 (GRP78; also known as BiP) in rodent beta cell lines^4^. GRP78 belongs to the heat-shock protein family and is abundantly expressed in all cell types. Its major subcellular location is the ER, where it plays a key role in protein folding. GRP78 expression is upregulated during the unfolded protein response (UPR), which is triggered in response to ER stress. The main mediators of the UPR are three transmembrane ER proteins, namely, activating transcription factor 6 (ATF6), protein kinase RNA-like ER kinase, and serine/threonine-protein kinase/endoribonuclease 1. ATF6 is the main regulator of GRP78 transcription^5,6^. Next to this well-studied function in the ER, GRP78 has also been observed in other subcellular locations, such as cytoplasm, mitochondria, nucleus, and plasma membrane, and was shown to be secreted into the extracellular space and present in the circulation^4,7^.

Secretion and translocation of GRP78 from the ER to the plasma membrane is associated with several pathological conditions, e.g. autoimmune diseases, such as rheumatoid arthritis^8^, and cancers, such as melanoma^9^ and prostate cancer^10^. Although the physiological function of cell surface GRP78 (sGRP78) is not fully elucidated, it has been shown that sGRP78 can act as a multifunctional signaling receptor interacting with various ligands and influencing processes, such as cellular proliferation, apoptosis, cell survival and metabolism^11^. In addition, secreted GRP78 might have immunogenic characteristics, against which the generation of autoantibodies has been reported^12^. How GRP78 translocates to and anchors in the plasma membrane and which signaling pathways are regulated by sGRP78 in stressed beta cells, remain to be clarified.

Here we report on cell surface translocation of GRP78 in rodent MIN6 cells, human EndoC-βH1 cells, and primary human islets; the underlying mechanism of GRP78 translocation; and the function of GRP78 on the beta cell plasma membrane. The mechanism of translocation involves the co-chaperone DNAJC3 and it is, at least in part, mediated through the Golgi complex and secretory vesicles. Blocking experiments with anti-GRP78 antibodies binding the N- or C-terminal domain of sGRP78 reveal that sGRP78 plays a prominent role in activating pro-apoptotic signaling cascades in beta cells. Together with our observation that extracellular, soluble GRP78 is a self-ligand for sGRP78, these results provide evidence for a novel pathway of active self-destruction in inflamed beta cells by setting up a pro-apoptotic self-destructive loop through the combined surface translocation and secretion of GRP78.

## Results

### Exposure to inflammatory cytokines induces surface translocation of GRP78 in beta cells

**Fig. 1:**
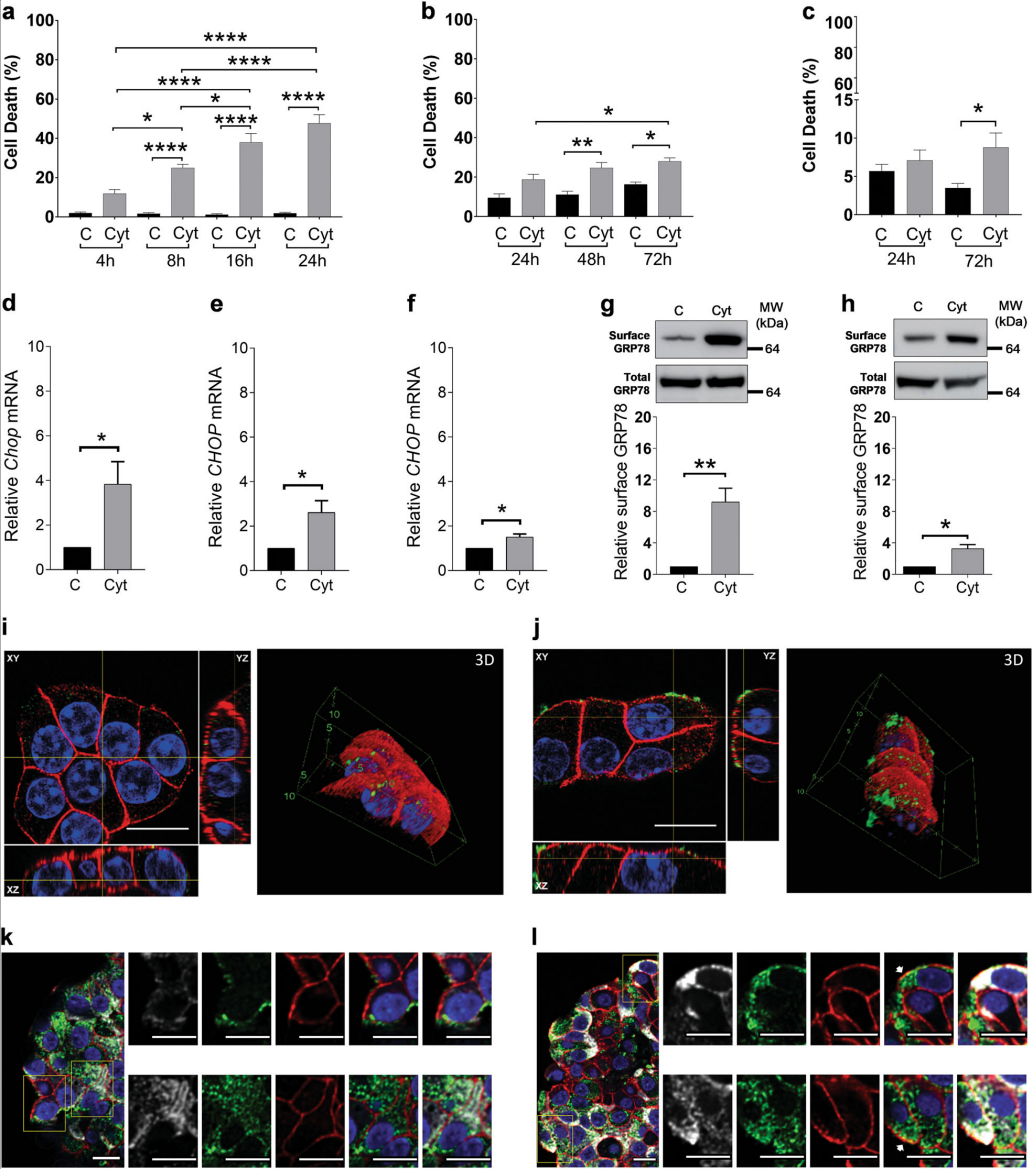
Exposure of beta cells to pro-inflammatory cytokines induces surface translocation of glucose-regulated protein 78 (GRP78). **a**–**c** Apoptosis levels in MIN6 exposed to human interleukin (hIL)-1β (50 U/ml), mouse interferon (mIFN)-γ (250 U/ml), and mouse tumor necrosis factor (TNF)-α (1000 U/ml) (*n* = 4) (**a**); EndoC-βH1 exposed to hIL-1β (50 U/ml), hIFN-γ (1000 U/ml), and mTNF-α (1000 U/ml) (*n* = 4) (**b**); and human islets exposed to hIL-1β (50 U/ml), hIFN-γ (1000 U/ml), and mTNF-α (1000 U/ml) (*n* = 8) (**c**) for the indicated time points. **d**–**f** *Chop* mRNA expression upon exposure to the same mix of cytokines in MIN6 (8 h, *n* = 4) (**d**), EndoC-βH1 (24 h, *n* = 5) (**e**), and human islets (24 h, n = 3) (**f**). **g**, **h** Surface and total GRP78 protein levels in MIN6 (**g**) and EndoC-βH1 (**h)** after 16 and 72 h cytokine exposure, respectively. Upper panels show one representative Western blot out of three independent experiments, performed on membrane fractions for surface GRP78 and on cytoplasmic fractions for total GRP78. Lower panels show the relative intensities of the different protein bands after quantification by densitometry and expressed as a ratio to the control. **i**, **j** Confocal images of live-cell staining of control (**i**) and cytokine-exposed (16 h) MIN6 cells (**j**), stained for GRP78 (green), the membrane marker β-catenin (red), and nuclei (blue). Per condition, one representative image out of three independent experiments is shown. **k**, **l** Confocal images of paraffin-embedded control (**k**) and cytokine-exposed (72 h) human islets (**l**), stained for GRP78 (green), the membrane marker β-catenin (red), insulin (white), and nuclei (blue). Per condition, one representative image out of five independent experiments is shown. Scale bars, 5 µm. Data are presented as mean ± SEM. Statistical analysis was performed by one-way analysis of variance for cell death and two-tailed Student’s *t* test for western blots. *P < 0.05, **P < 0.01, and ****P < 0.0001

Exposure to a mixture of the pro-inflammatory cytokines interleukin (IL)-1β, interferon (IFN)-γ, and tumor necrosis factor (TNF)-α induced apoptosis in murine MIN6 cells (Fig. 1a), in human EndoC-βH1 cells (Fig. 1b), and in primary human islets (Fig. 1c). This phenomenon was preceded by ER stress activation, as evidenced by the early induction of the pro-apoptotic ER stress marker *CHOP* (Fig. 1d–f). Western blot analysis of membrane fractions, obtained from control and cytokine-exposed MIN6 and EndoC-βH1 cells, demonstrated a nine- and three-fold increase of sGRP78 following cytokine exposure, respectively, whereas total GRP78 levels were not altered (Fig. 1g, h). We confirmed these findings using live-cell staining techniques, observing enhanced GRP78 membrane co-expression with cell surface marker β-catenin upon cytokine exposure in MIN6 cells (Fig. 1i, j). Similarly, co-localization of GRP78 with β-catenin was observed in insulin-positive cells in cytokine-exposed human islets, providing evidence that the mechanism of translocation also takes place in primary human islets (Fig. 1k, l). These findings support our earlier published findings in rat INS-1E cells and murine islets^4^, suggesting that cytokine-induced GRP78 translocation is a general mechanism taking place in rodent and human beta cell lines, as well as in rodent and human primary islet cells.

### Surface translocation of GRP78 in beta cells is an early response to inflammation

**Fig. 2:**
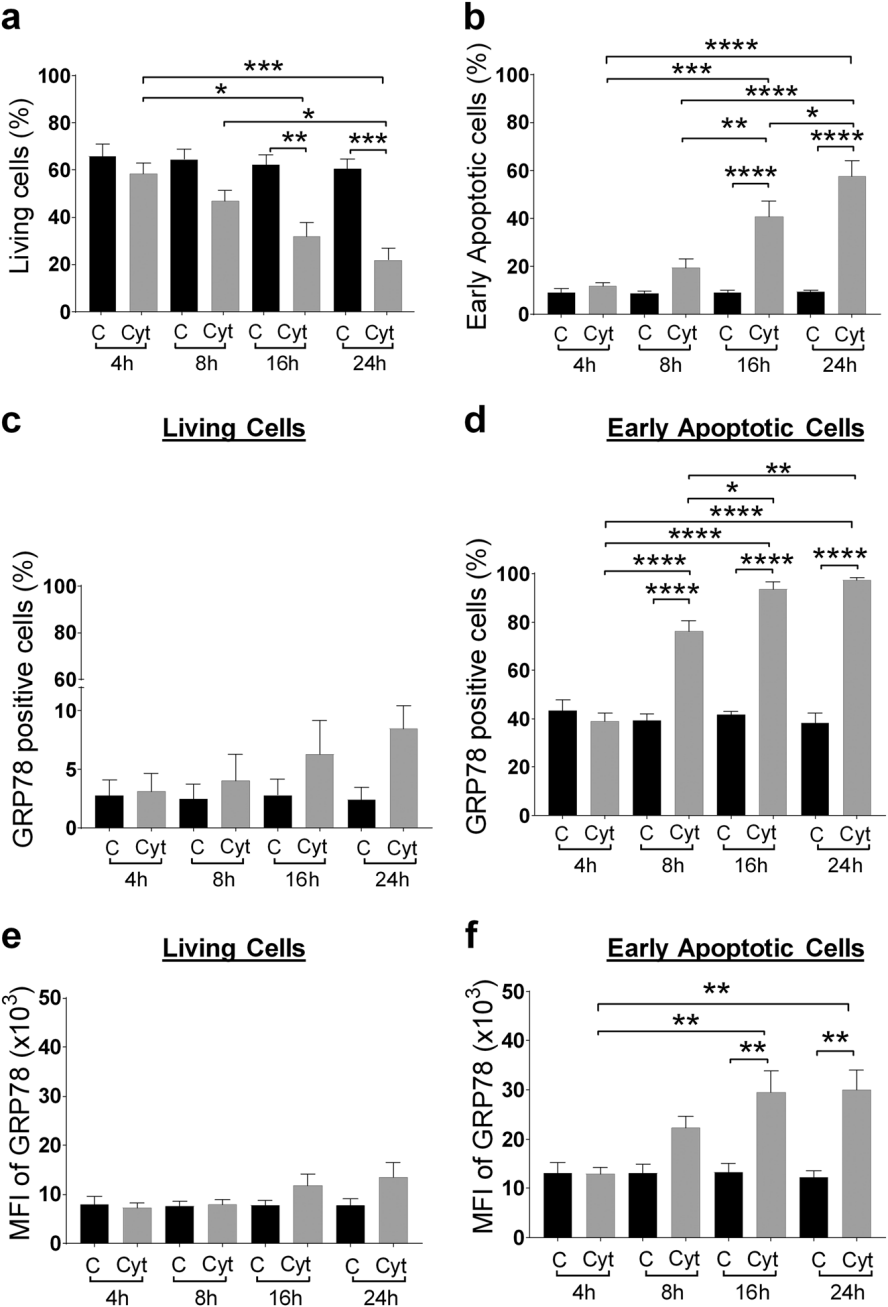
Surface translocation of glucose-regulated protein 78 (GRP78) in beta cells is an early phenomenon, taking place in the early apoptotic cell population. MIN6 cells exposed to cytokines (human interleukin-1β (50 U/ml), mouse interferon-γ (250 U/ml), and mouse tumor necrosis factor-α (1000 U/ml)) for the indicated time points (4, 8, 16, and 24 h) were stained with the late-apoptosis/necrosis marker DRAQ7-AF700, early apoptosis marker Annexin V-APC (see Results for the interpretation of these combined dyes to discriminate between living, early, and late apoptotic cells), and anti-GRP78-FITC, followed by flow cytometry. **a**, **b** Percentage of living (**a**) and early apoptotic (**b**) cell population in control (C) and cytokine (Cyt) exposed MIN6 cells. **c**, **d** Percentage of sGRP78-positive cells in the living (**c**) and early apoptotic (**d**) subpopulation. **e**, **f** Geometric mean fluorescence intensity (MFI) for sGRP78 in the living (**e**) and early apoptotic (**f**) subpopulation. Data are presented as mean ± SEM (*n* = 4) and statistically analyzed by a two-way analysis of variance followed by Sidak posthoc test for multiple group comparisons. *P < 0.05, **P < 0.01, ***P < 0.001, and ****P < 0.0001

We next examined the time course of cytokine-induced translocation of GRP78 to the plasma membrane in murine MIN6 cells by flow cytometry as compared to markers of cell death (Fig. 2). Surface exposure of phosphatidylserine (PS) precedes apoptosis-associated membrane rupture^13^, and we used the PS-binding protein annexin V and the cell-impermeable DNA dye DRAQ7 to discriminate between living (annexin V negative, DRAQ7 negative), early apoptotic (annexin V positive, DRAQ7 negative), and late apoptotic/necrotic (DRAQ7 positive) cell populations (Fig. S1). We selected a time window in which the living population clearly transitions to the early apoptotic cell population but without a notable increase in the late apoptotic/necrotic cell population, thus enabling analysis on cells with intact membranes. The percentage of living and early apoptotic cells did not significantly change in the first 8 h of cytokine exposure (Fig. 2a, b). Interestingly, at this early time point, a significantly higher percentage of the early apoptotic cell population expressed surface-bound GRP78 after cytokine exposure (cytokine-treated: 76 ± 4% vs. control: 39 ± 2%, p < 0.0001) (Fig. 2d), indicating that surface expression of GRP78 is an early response to inflammation. This percentage of GRP78-positive cells further increased until a plateau was reached 16 h after cytokine exposure, at which 94 ± 2% of the early apoptotic cells expressed GRP78 on their cell membrane. Furthermore, flow cytometry indicated that the GRP78-positive cell fraction had a greater mean fluorescence intensity (MFI) upon cytokine exposure, indicative for elevated sGRP78 expression (Fig. 2f). A similar trend could be observed in the living cell population, but this did not reach statistical significance (Fig. 2c, e).

### Membrane translocation of GRP78 in beta cells is, in part, Golgi dependent

**Fig. 3:**
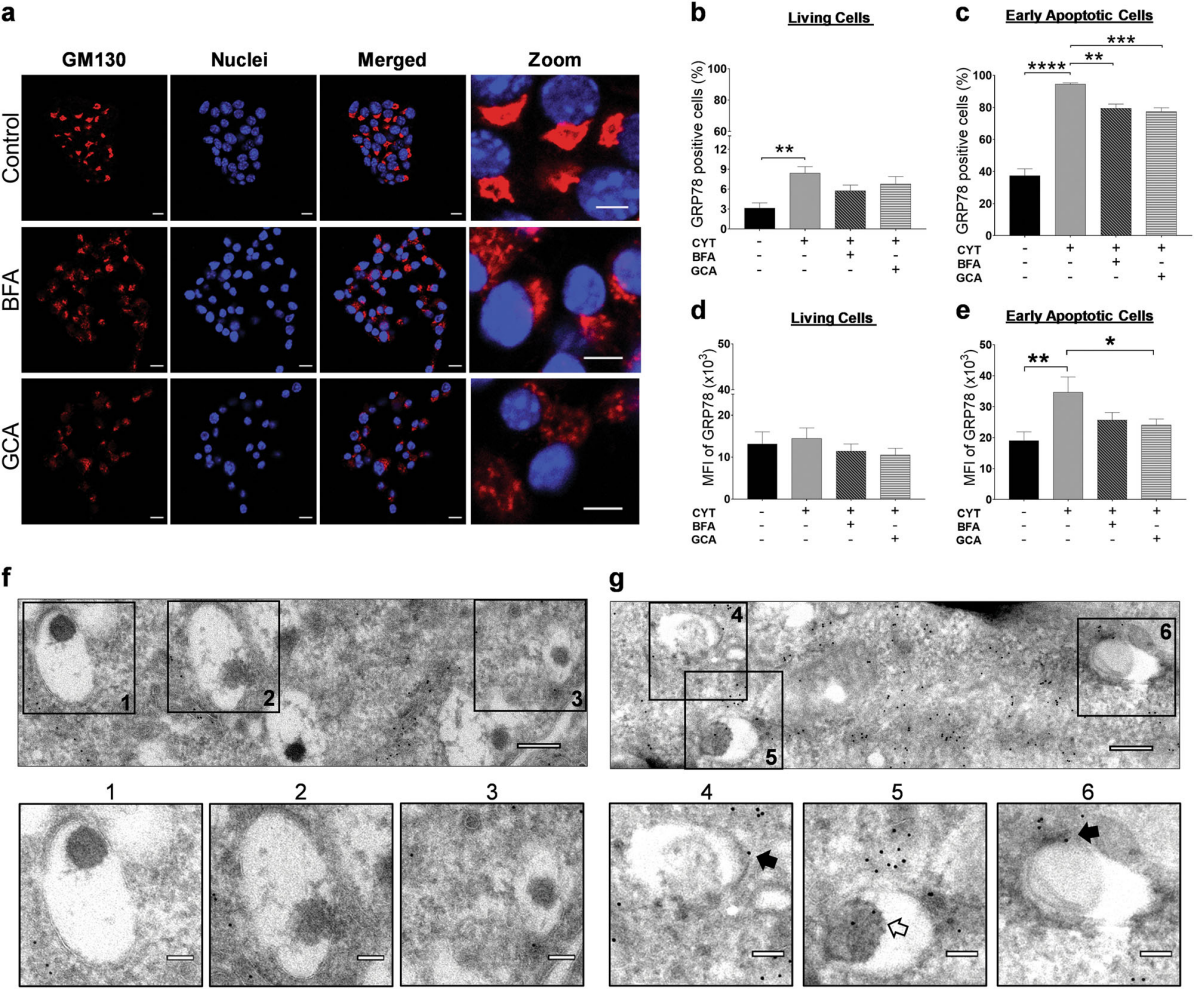
Cytokine-induced glucose-regulated protein 78 (GRP78) membrane translocation is Golgi and secretory pathway dependent. **a** Microscopic imaging of Golgi apparatus disruption with brefeldin A (BFA; 0.2 μg/ml, 16 h) and golgicide A (GCA; 10 μM, 16 h) in MIN6 cells, evaluated by immunostaining with the Golgi marker 130 (GM130; red) and Hoechst (Hoechst 33342; blue). One representative experiment out of three independent experiments is shown. Scale bars, 10 μm (without zoom) and 5 μm (with zoom). **b**–**e** Flow cytometry of MIN6 cells upon co-incubation of cytokines (Cyt) with BFA or GCA. Percentage of sGRP78-positive cells in the living (**b**) and early apoptotic (**c**) subpopulation. MFI for surface GRP78 in the living (**d**) and early apoptotic (**e**) subpopulation. Data are presented as mean ± SEM (*n* = 7) and statistically analyzed by a one-way analysis of variance followed by Sidak posthoc test for multiple group comparisons. *P < 0.05, **P < 0.01, ***P < 0.001, and ****P < 0.0001. **f**, **g** Transmission electron microscopic (TEM) images of Immunogold-labeled GRP78 in control (**f**) and cytokine-exposed (16 h) (**g**) MIN6 cells. Per condition, one representative image out of three independent experiments is shown. Black arrows point to the GRP78-bound gold particle (10 nm) localized in the secretory granules’ limiting membranes, whereas white arrows indicate GRP78-bound gold particles localized within the secretory vesicles. Images were captured at ×10,000 zoom. Scale bars are 250 nm in the upper panel and 100 nm in the lower panel

To determine whether cytokine-induced translocation of GRP78 to the cell surface follows the traditional secretory ER-to-Golgi traffic, we assessed the effect of brefeldin A (BFA; 0.2 µg/ml) or golgicide A (GCA; 10 μM), compounds that block protein transport from the ER to the Golgi apparatus^14,15^, on this process. Both compounds effectively caused Golgi dispersal, shown by the fragmented localization of the cis-Golgi matrix protein GM130 (Fig. 3a). The percentage of GRP78-positive cytokine-exposed beta cells in the early apoptotic subpopulation was significantly reduced in the presence of BFA or GCA (Fig. 3b, c). Similarly, MFI profiles of GRP78-positive cytokine-exposed beta cells showed reduced GRP78 expression when co-incubated with BFA or GCA (Fig. 3d, e). We next performed immunogold transmission electron microscopy to visualize GRP78 expression in secretory granules. GRP78 was more abundantly present after cytokine exposure (Fig. 3g) as compared to control cells (Fig. 3f), with indications of its presence in secretory granules (open arrows) as well as on the granules’ limiting membranes (closed arrows). Together, these results imply that GRP78 membrane translocation in beta cells is, in part, Golgi dependent by trafficking through the secretory granules.

### DNAJC3 facilitates GRP78 translocation to the plasma membrane in beta cells

**Fig. 4:**
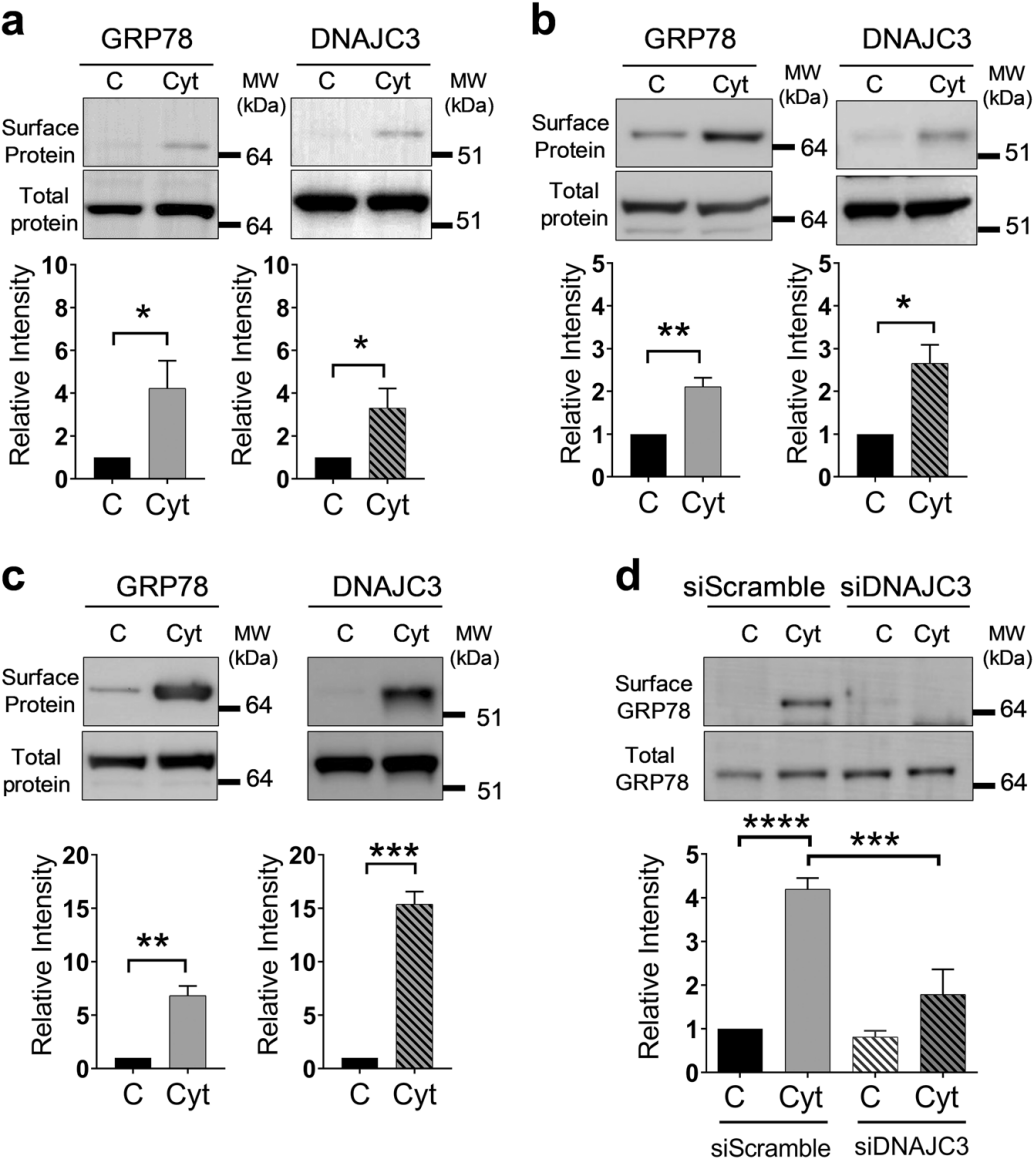
DNAJC3 acts as co-transporter for glucose-regulated protein 78 (GRP78) surface translocation. **a**–**c** GRP78 and DNAJC3 protein expression on the surface and total protein by western blots in INS-1E exposed to human interleukin (hIL)-1β (50 U/ml) and rat interferon (rIFN)-γ (500 U/ml) (16 h; *n* = 3) (**a**); EndoC-βH1 exposed to hIL-1β (50 U/ml), hIFN-γ (1000 U/ml), and mouse tumor necrosis factor (mTNF)-α (1000 U/ml) (24 h; *n* = 3) (**b**); and MIN6 exposed to hIL-1β (50 U/ml), mIFN-γ (250 U/ml), and mTNF-α (1000 U/ml) (16 h; *n* = 3) cells (**c**). **d** Surface and total GRP78 protein levels in MIN6 transfected with control scramble (10 nM) and DNAJC3 (10 nM) small interfering RNA for 48 h and exposed to cytokines for 16 h (*n* = 4). Upper panels show one representative western blot out of four independent experiments. Lower panels show the relative intensities of the different protein bands after quantification by densitometry and expressed as a ratio. Data are presented as mean ± SEM and statistically analyzed by two-tailed unpaired Student’s *t* test. Significance is indicated by *P < 0.05, **P < 0.01, ***P < 0.001, and ****P < 0.0001

GRP78 translocation in macrophages is facilitated via its interaction with murine tumor cell DNAJ-like protein 1 (MTJ-1), a DNAJ-like transmembrane protein^16^. To investigate whether sGRP78 is also accompanied or facilitated by co-chaperones in beta cells, we isolated membrane fractions from control and cytokine-exposed INS-1E cells by immunoprecipitation with an anti-GRP78 antibody. Subsequent liquid chromatography-tandem mass spectrometry (LC-MS/MS) analysis of the immunoprecipitate identified 61 unique sGRP78-interacting proteins in cytokine-exposed INS-1E cells (Fig. S2a, b and Table S3). Interestingly, among these proteins we detected DNAJC3, previously described as a co-chaperone of GRP78 in the ER^17^. Western blot analysis of membrane protein lysates demonstrated that the cytokine-induced surface translocation of GRP78 was paralleled by an increase in DNAJC3 surface expression in INS-1E and EndoC-βH1 as well as in MIN6 cells (Fig. 4a–c). Silencing of *DNAJC3* gene expression in MIN6 cells using a specific small interfering RNA (siRNA; 60% knockdown compared to scrambled siRNA (Fig. S2c)) reduced the cytokine-induced sGRP78 protein expression by more than two-fold (Fig. 4d). These results demonstrate that DNAJC3 is required for cell surface trafficking of GRP78 in beta cells.

### Cell sGRP78 has a pro-apoptotic role in beta cells and induces ER stress

**Fig. 5:**
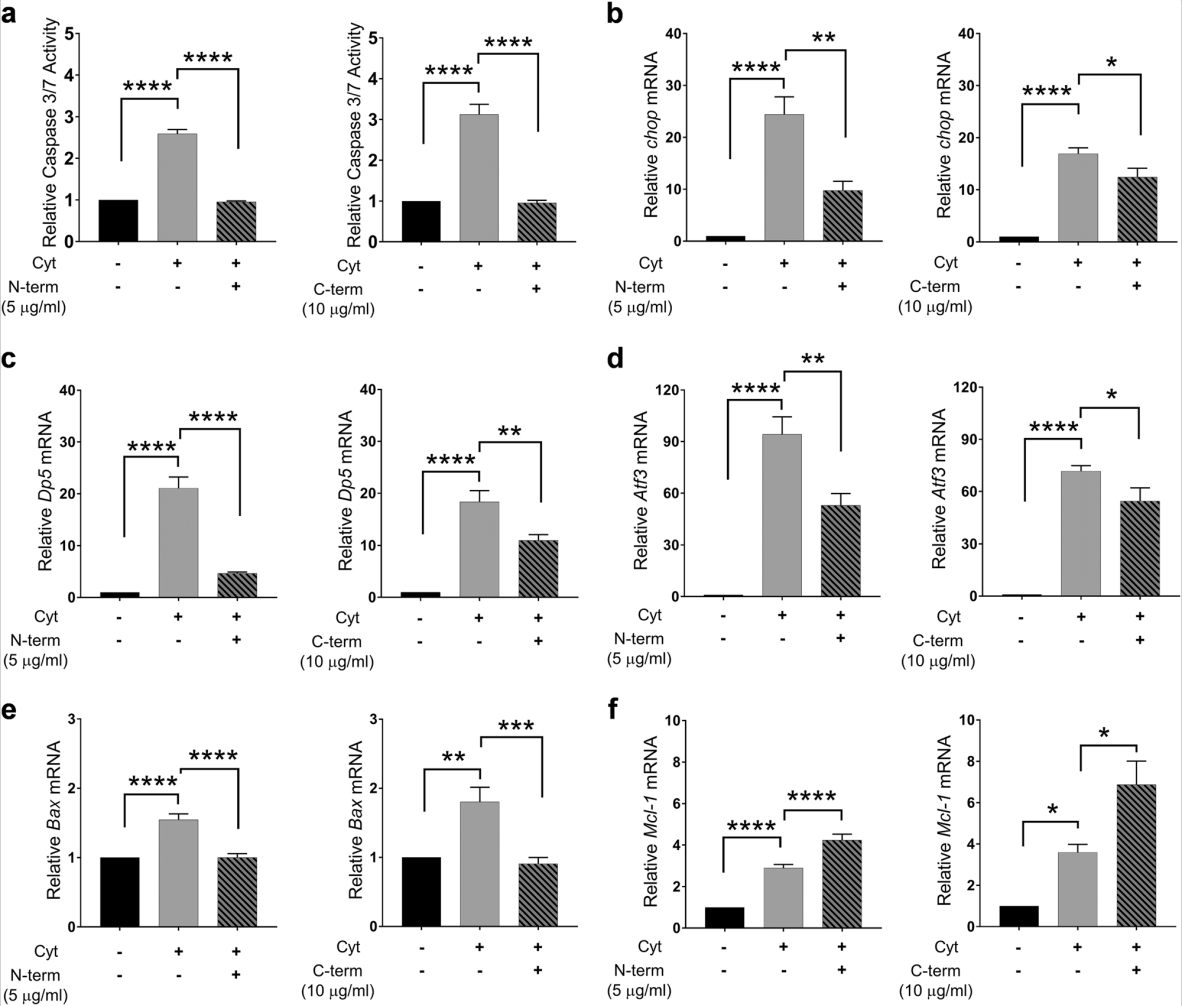
Surface glucose-regulated protein 78 (GRP78) acts as a pro-apoptotic signaling receptor. **a** Relative caspase 3/7 activity in INS-1E cells exposed to cytokines (human interleukin (hIL)-1β (50 U/ml) and rat interferon (rIFN)-γ (500 U/ml)) with or without the addition of anti-GRP78 antibody against the N-terminal region (N-term, 5 μg/ml, left panel. *n* = 5) or C-terminal region (C-term, 10 μg/ml, right panel, *n* = 5) for 16 h. **b**–**f** Relative mRNA expression of pro-apoptotic markers *Chop* (**b**), *Dp5* (**c**), *Atf3* (**d**), and *Bax* (**e**) and the anti-apoptotic marker *Mcl-1* (**f**) in control and cytokine-exposed INS-1E cells (hIL-1β (50 U/ml), rIFN-γ (500 U/ml)) with or without the addition of anti-GRP78 antibody against the N-terminal (N-term, 5 μg/ml, left panels, *n* = 3) or C-terminal (C-term, 10 μg/ml, right panels, *n* = 5) region for 16 h. Data are presented as mean ± SEM and statistically analyzed by a one-way analysis of variance followed by Sidak posthoc test for multiple group comparisons. Significance is indicated by *P < 0.05, **P < 0.01, ***P < 0.001, and ****P < 0.0001

To establish the putative functional role of sGRP78 as a membrane receptor in beta cells, we used antibodies that specifically block the C- and N-terminal regions of the protein. Co-incubation of cytokine-exposed INS-1E cells with either of these antibodies significantly reduced cytokine-induced apoptosis and cellular dysfunction, as evaluated by caspase 3/7 activation (Fig. 5a) and *Ins2* transcription levels (Fig. S4), respectively, whereas incubation with relevant control IgG did not have an effect (data not shown). Subsequent analysis of pro- and anti-apoptotic transcription factors demonstrated attenuated expression of the pro-apoptotic markers *Chop* (Fig. 5b), *Dp5* (Fig. 5c), *Atf3* (Fig. 5d), and *Bax* (Fig. 5e) and enhanced expression of the anti-apoptotic marker *Mcl-1* (Fig. 5f), following blocking the C- and N-terminal regions of sGRP78. These results suggest that sGRP78 functions as a pro-apoptotic receptor in beta cells and that the underlying mechanism is mediated, at least in part, by ER stress.

### Extracellular GRP78 is a self-ligand for sGRP78 in beta cells and activates pro-apoptotic signaling pathways

**Fig. 6:**
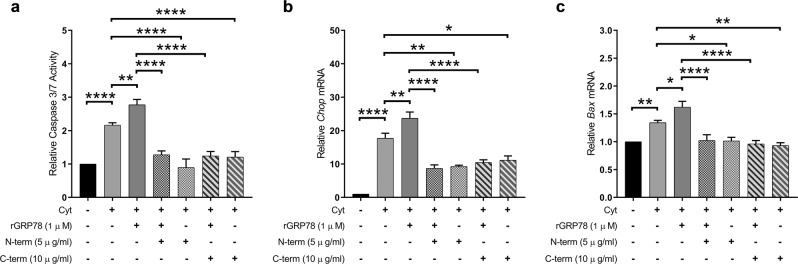
Soluble glucose-regulated protein 78 (GRP78) increases cell death and endoplasmic reticulum stress. **a**–**c** Relative caspase 3/7 activity (**a**) and relative mRNA expression of *Chop* (**b**) and *Bax* (**c**) in cytokine-exposed INS-1E cells (hIL-1β (50 U/ml), rat interferon-γ (500 U/ml)) in the presence of recombinant GRP78 (1 μM) with or without anti-GRP78 antibodies against the N-terminal region (N-term, 5 μg/ml) or C-terminal region (C-term, 10 μg/ml) for 16 h (*n* = 4). Data are presented as mean ± SEM and statistically analyzed by a one-way analysis of variance followed by Sidak posthoc test for multiple group comparisons. Significance is indicated by *P < 0.05, **P < 0.01, and ****P < 0.0001

sGRP78 can act as a membrane receptor for extracellular, soluble GRP78, as reported in the colorectal adenocarcinoma cell line DLD1^18^. Given our previous observations that the secretion of GRP78 by INS-1E cells is significantly enhanced when exposed to pro-inflammatory cytokines^4^, we assessed the ability of extracellular, soluble GRP78 to activate sGRP78 by incubating INS-1E cells with recombinant GRP78. In vitro addition of recombinant GRP78 enhanced apoptosis in cytokine-exposed INS-1E cells in a dose-dependent manner (Fig. S3 and Fig. 6a), whereas it had no significant effect on control INS-1E cells (data not shown), suggesting that enhanced sGRP78 expression is a prerequisite for recombinant GRP78 to act. Interestingly, cytokine-induced ER stress, as evaluated by *Chop* and *Bax* mRNA expression, was further enhanced by addition of recombinant GRP78 to the culture medium (Fig. 6b, c). Co-incubation with anti-GRP78 antibodies blocking either the N- or C-terminal end of sGRP78 decreased the induction of pro-apoptotic signaling pathways, as evidenced by reduced caspase 3/7 activation (Fig. 6a) and attenuated *Chop* (Fig. 6b) and *Bax* transcription (Fig. 6c). A similar attenuating effect was observed for the pro- and anti-apoptotic transcription factors *Dp5*, *Atf3*, and *Mcl-1* (Fig. S5). Together, these results suggest that extracellular GRP78 is able to bind to sGRP78 and by this mechanism promotes beta cell ER stress and apoptosis.

## Discussion

We presently provide the first evidence for a novel pathway of active self-destruction in cytokine-exposed beta cells through the ER chaperone GRP78. We demonstrate that beta cells not only secrete GRP78 when exposed to inflammatory stress^4^ but that they also translocate GRP78 to the cell surface where it acts as a pro-apoptotic signaling receptor. Together with our observation that soluble GRP78 can act as a ligand and activator of sGRP78, these results indicate that beta cells trigger a self-destructive feedback loop when exposed to inflammation, actively participating in their own demise.

Previously, we have shown that GRP78 is translocated to the plasma membrane of rat INS-1E cells and murine islets when exposed to pro-inflammatory cytokines^4^. We now extend these observations to cytokine-exposed murine and human beta cell lines and to primary human islet cells. We demonstrate that GRP78 translocation is a rapid response to inflammation, with enhanced sGRP78 expression detected already 8 h after cytokine exposure in early apoptotic cells with intact membranes. Intriguingly, surface translocation of GRP78 in response to stress is not limited to beta cells. Several studies have reported that the chemical ER stressors thapsigargin and tunicamycin substantially increase GRP78 surface expression in various cell lines, such as 293T human embryo kidney fibroblast cells^19^ and the human cancer cell lines HeLa^20^, MCF-7^20^, HCT-116^20^, and TE671/RD^21^, which is in line with our previous observations in beta cells that not only cytokines but also thapsigargin induces translocation of GRP78^4^. These observations suggest that surface translocation of GRP78 may be secondary to a (patho)physiological process such as ER stress.

The mechanisms by which GRP78 is translocated to the cell surface have not been fully unraveled, with divergent findings regarding the subcellular route of translocation indicating that different cell types may use different routes in this process^20^. We show here that the trafficking route of GRP78 to the membrane of beta cells is in part Golgi dependent and mediated via secretory granules. Such a route of translocation may explain its secretion into the extracellular milieu^4^ and is consistent with the detection of GRP78 in serum^22^ or spinal fluid^23^ of human subjects. Moreover, we have recently shown that a subpopulation of human subjects have circulating autoantibodies against GRP78, with enhanced levels in subjects with T1D^24^. However, our present observation that the secretory pathway inhibitors BFA and GCA do not completely block the surface translocation implicates that additional mechanisms are involved. Chaperones are typically retained within the ER via a retrograde transport pathway mediated by their KDEL sequence. One possibility is that interacting proteins mask this KDEL sequence, allowing the chaperones to escape the retrograde retrieval system^25^. In this regard, it has been shown in macrophages that GRP78-interacting protein partners can assist in the membrane translocation of GRP78^16,26^. Here, we identified DNAJC3, a co-chaperone of GRP78 in the ER^27^, as a GRP78 interacting protein partner. In macrophages, GRP78 interacts with the J domain of another DNAJ homolog subfamily C member, DNAJC1 (also known as MTJ1)^16^. Interestingly, this J domain is preserved in all DNAJ-like proteins^26^, therefore establishing DNAJ-like family members as candidate proteins capable of interacting with GRP78. The exact route taken by these interacting protein partners to facilitate GRP78 translocation to the surface remains to be elucidated, although there is some evidence that these retrograde pathways go through the cytosol^12^. If this would be true, this could explain our previous observations that GRP78 is citrullinated in the cytosol of stressed beta cells^4,24^, as the peptidylarginine deiminase enzymes responsible for this posttranslational modification are mainly located in the cytosol^28^.

In this study, we show that sGRP78 has a prominent pro-apoptotic function in cytokine-exposed beta cells. Intriguingly, in tumor cells sGRP78 is associated with enhanced proliferation^11,29^. It has been suggested that this difference in physiological response may depend on whether sGRP78 is engaged at its N- or C-terminus. sGRP78 contains four hydrophobic domains, which are predicted to form transmembrane helices localizing both the N- and C-terminal regions outside of the membrane^19^. By using N- and C-terminal blocking antibodies, it has been shown that sGRP78 can signal via these two compartments, leading to different and sometimes even opposing effects^11^. In cancer cells, ligation of sGRP78 with blocking antibodies against the N-terminal domain induces proliferation^10^, whereas ligation with blocking antibodies against the C-terminal domain promotes apoptosis^30–32^. By using similar blocking antibodies, we presently demonstrate that in beta cells both the N- and C-terminal domain of sGRP78 activate pro-apoptotic signaling cascades. This observation suggests that in beta cells the function of sGRP78 as a signaling receptor does not depend on its site of activation.

We further demonstrate that activation of the sGRP78 receptor by recombinant GRP78, a ligand of sGRP78^18^, induces activation of pro-apoptotic signaling pathways, evidenced by increased caspase activity and cell death, which is paralleled by enhanced *Chop* and *Bax* expression. This GRP78-induced caspase activation could be blocked by co-incubation with both N- and C-terminal blocking antibodies. In DLD1 cells, the opposite effect of recombinant GRP78 as a ligand for sGRP78 has been reported^18^, in line with the contrasting function of the N-terminal end of sGRP78 in cancer as compared to beta cells.

As both signaling domains of sGRP78 initiate pro-apoptotic pathways when activated in beta cells, sGRP78 may thus be an attractive target for beta cell-preserving therapies in T1D. The ideal therapeutic compound should either prevent GRP78 surface translocation or suppress the GRP78 receptor signaling, while preserving the GRP78 chaperone function in the ER. Over the past few years, several GRP78 activators, inhibitors, and blocking antibodies have been developed, regulating proliferation and/or apoptosis^33–36^, but their effect on inflamed beta cells remains to be evaluated.

Taken together, we demonstrate here for the first time that sGRP78 serves as a pro-apoptotic signaling receptor in beta cells. Based on our previous observations that cytokine-exposed beta cells secrete GRP78^4^ and that soluble GRP78 is a ligand for sGRP78, thereby activating its apoptotic receptor function, we postulate that inflamed beta cells set up a self-destructing feedback loop through the combined surface translocation and secretion of GRP78. This makes sGRP78 an important modulator of beta cell death upon inflammatory stress responses. Targeting sGRP78 in T1D may thus be a promising therapeutic strategy for beta cell preservation.

## Materials and methods

### Cell lines, human islets, and culture conditions

Rat INS-1E cells, a kind gift from Professor C. Wollheim (Centre Medical Universitaire, Geneva, Switzerland), were cultured in RPMI 1640 medium (#61870010; Invitrogen, CA, USA) supplemented with 10 mmol/l HEPES, 10% (v/v) heat-inactivated fetal bovine serum (FBS; #F7524, Sigma, MO, USA), 100 U/ml penicillin and 100 μg/ml streptomycin (1× P/S; #15140122; Invitrogen), 1 mmol/l sodium pyruvate, and 50 μM β-mercaptoethanol^37^. INS-1E cells were used from passage 64 to 70. Mouse insulinoma (MIN6) cells, a kind gift from Dr. Miyazaki (Osaka University, Japan), were grown in Dulbecco's Modified Eagle Medium (DMEM; #21969–035, Invitrogen) supplemented with 15% FBS, 1× P/S, 4 mM glutamax (#35050038; Invitrogen), and 70 µM β-mercapthoethanol^38^. MIN6 cells were used from passage 25 until 33. Human EndoC-βH1 cells, a kind gift from R. Scharfmann (Centre de Recherche de l’Institut du Cerveau et de la Moelle Épinière, Paris, France)^39^, were used from passage 61 until passage 80. Culture plates were coated with Matrigel-fibronectin. Human EndoC-βH1 cells were cultured in DMEM (#31885023; Invitrogen), containing 2% bovine serum albumin (BSA), 10 mM nicotinamide, 5.5 μg/ml transferrin, 6.7 ng/ml sodium selenite, 50 μM β-mercaptoethanol, and 1× P/S. Absence of Mycoplasma contamination in all cell lines was confirmed by PCR analysis using the Venor®GeM Mycoplasma PCR Detection Kit (# 11–91100, Minerva Biolabs, Berlin, Germany).

Primary human islets were obtained from the Alberta Diabetes Institute Islet Core, with the agreement of the local Ethical Committee from the University of Alberta (Pro00013094; Pro00001754)^40,41^. They were cultured in DMEM, supplemented with 1% l-glutamine (# 25030081, ThermoFisher scientific, MA, USA), 10% FBS, and 100 U/ml 1× P/S. The characteristics of islet donors are provided in Supplementary Table S1.

INS-1E cells were exposed to recombinant rat IFN-γ (# 585-IF-100, 500 U/ml; R&D Systems, Minneapolis, MN, USA) and human IL-1β (# 201-LB-005, 10 U/ml; hIL-1β; R&D Systems). MIN6 cells were exposed to hIL-1β (50 U/ml), mouse IFN-γ (# 315–05, 250 U/ml; mIFN-γ; PeproTech, London, UK) and mouse TNF-α (# 315-01A, 1000 U/ml; mTNF-α; PeproTech) for the indicated time points (see figure legends). EndoC-βH1 cells were exposed to hIL-1β (50 U/ml), human IFN-γ (# 11 040 596 001, 1000 U/ml hIFN-γ; Preprotech), and mTNF-α (1000 U/ml) for the indicated time points (see figure legends). Human islets were exposed to hIL-1β (50 U/ml), mTNF-α (1000 U/ml), and hIFN-γ (1000 U/ml) for the indicated time points (see figure legends). Lower cytokine concentrations and shorter incubation times were used in the INS-1E and MIN6 experiments, because rodent beta cells are more sensitive to cytokine-induced damage than human beta cells^4,42–44^.

### Cell viability assays

The percentage of apoptosis in beta cell lines and in human islets was determined using propidium iodide (#P3566; Invitrogen) and Hoechst 33342 (#H3570; Invitrogen)^45^. In addition, apoptosis was analyzed by measuring caspase-3/7 activity using the Caspase-Glo® 3/7 Assay (# G8091, Promega, WI, USA), according to the manufacturer’s instructions.

### Flow cytometry

Cells were harvested using accutase (#00-4555-56, ThermoFisher scientific), followed by Fc-receptor blocking with anti-CD16/32 (#14-0161-85; ThermoFisher scientific; 1:100, 10 min at 4 °C) prior to staining. Single-cell suspensions were stained with anti-GRP78-A488 (#PA1-014A-A488; ThermoFisher scientific; 1:400, 20 min at 4 °C), APC-Annexin V (#640941; Biolegend, San Diego, CA, USA; 1:20, 15 min at room temperature (RT)), and DRAQ7 (#424001; Biolegend; 1:400, 10 min at RT). Analysis was performed on a Gallios^TM^ Beckman Coulter flow cytometer (Beckman Coulter, CA, USA) with the FlowJo software (FlowJo, LLC, Ashland, OR, USA).

### Immunofluorescence

For surface staining of sGRP78, MIN6 cells (0.8 × 10^6^) were washed with ice-cold phosphate-buffered saline (PBS; pH 7.4) and incubated with anti-GRP78-A488 (#PA1-014A-A488; ThermoFisher scientific; 1:100) antibody for 30 min at 4 °C in the dark. After incubation, the cells were washed and fixed with ice-cold methanol (100%) for 10 min at 4 °C. Blocking was performed in 0.4% fish gelatin, 1% BSA, and 0.1% triton-X 100 in PBS (30 min at RT). Thereafter, samples were incubated with mouse-anti-β-catenin (#2677S; Cell Signaling, MA, USA, 1:100) antibody for 1 h at RT, followed by washing with PBS and incubation with the fluorophore-conjugated secondary antibody (Alexa Fluor® 647-conjugated goat anti-mouse; #A-21235; ThermoFisher scientific) for 1 h at RT. Nuclei were stained with Hoechst 33342 (10 μg/ml) (Invitrogen). Specificity was confirmed by including negative controls with secondary antibodies alone. *Z*-stacks with a step size of 150 nm were acquired using a laser scanning confocal microscope (Leica TCS SP8 STED, ×100 1.4 NA oil objective, Leica Microsystems, Mannheim, Germany). Per plane, channels were collected sequentially (512 × 512 pixels). Images were post-processed using the Huygens deconvolution software version 17.04 (Huygens, Hilversum, the Netherlands). Three-dimensional rendering was performed in ImageJ/Fiji (http://rsb.info.nih.gov/ij/).

Primary human islets were washed in PBS and fixed with Z-fix (10% zinc formalin (pH5.5), # 170, Anatech, Battle Creek, MI, USA) overnight at 4 °C. The islets were then embedded in 2% (w/v) agarose and prepared for paraffin embedding. The samples were sectioned at 4 μm, deparaffinized in xylene, and rehydrated through graded ethanol series to distilled water. The sections were incubated with blocking solution (1% BSA in PBS). Primary antibodies used were rabbit anti-β-catenin (#8480S; Cell Signaling; overnight at 4 °C), mouse anti-GRP78 (#sc-376768; Santa Cruz Biotechnology, CA, USA, 1 h at RT), and guinea pig anti-insulin (#A-0564; Dako, Glostrup, Denmark, 1 h at RT). Alexa Fluor® 568-conjugated goat anti-rabbit (#A-11011; ThermoFisher Scientific, 1 h at RT), Alexa Fluor® 488-conjugated goat anti-mouse (#A11029; ThermoFisher Scientific, 1 h at RT), and Alexa Fluor® 647- conjugated goat anti-guinea pig (#706-605-148; Jackson ImmunoResearch, West Grove, USA, 1 h at RT) were used as secondary antibodies. Imaging was performed using a Zeiss LSM 780 microscope (×63, 1.15 NA, oil objective) with the ZEN 2 (Blue edition) software (Carl Zeiss AG, Jena, Germany). Channels were collected sequentially (1024 × 1024 pixels). Images were post-processed using the Huygens deconvolution software version 17.04 (Huygens, Hilversum, The Netherlands).

### Cell surface biotinylation and membrane fractionation

MIN6, INS-1E, and EndoC-βH1 cells (6 × 10^6^) were incubated as indicated in figure legends and were rinsed 3 times with ice-cold PBS containing 1 mM MgCl_2_ and 0.1 mM CaCl_2_. Thereafter, the cell surface proteins were labeled with biotin using EZ-Link Sulfo-NHS-SS-biotin (1.25 mg/ml; # 21331, ThermoFisher scientific) dissolved in biotinylation buffer (10 mM triethanolamine, 2 mM CaCl_2_, 150 mM NaCl, pH 7.5) for 30 min at 4 °C with slow agitation. Next, the cells were rinsed once with ice-cold PBS with 1 mM MgCl_2_ and 0.1 mM CaCl_2_ and incubated with quenching buffer (100 mM glycine in PBS) for 20 min at 4 °C under slow agitation. Subsequently, cells were rinsed twice with ice-cold PBS and solubilized in lysis buffer (10% glycerol, 1.0% Triton-X-100, 150 mM NaCl, 5 mM EDTA, 50 mM HEPES; pH 7.4) supplemented with Complete Mini protease inhibitor mixture (#11697498001; Sigma). The cleared lysate (150 μg) was then incubated for 16 h with streptavidin-agarose beads (#S951; Invitrogen) at 4 °C. After centrifugation, removal of the supernatant, and three washing steps in washing buffer (20 mM HEPES; pH 7.4, 150 mM NaCl, 10% glycerol and 0.1% Triton X-100), the biotinylated proteins were eluted by boiling the beads for 5 min in sodium dodecyl sulfate-polyacrylamide gel electrophoresis (SDS-PAGE) sample buffer with 5% β-mercaptoethanol. The resulting samples were subjected to SDS-PAGE and western blotting^46^.

### SDS-PAGE and western blotting

Protein lysates were separated on 4–12% Bis-Tris gels (# NP0321BOX, Invitrogen), blotted onto a polyvinylidene difluoridemembrane (# RPN132D, Hybond-ECL; GE Healthcare, Chicago, IL, USA), and probed with the indicated primary and secondary antibodies. The blots were incubated with Western lightning^TM^ Plus-ECL detection system (PerkinElmer, Waltham, MA, USA) and protein bands were quantified using ImageQuant LAS image 500 instrument (GE Healthcare).

### Immuno-gold staining and transmission electron microscopy

MIN6 cells were fixed with 0.2% glutaraldehyde (GA) in 0.1 M phosphate buffer (PB) pH 7.4. After centrifugation, pelleted cells were embedded in 12% gelatin in 0.1 M PB. After solidification, the pellets in gelatin were cut in small cubes and incubated in 2.3 M sucrose overnight and frozen on pins for cryosectioning. Seventy-nm sections were deposited on glow-discharged, carbon-coated 400 mesh copper grids. The grids were incubated in 2% gelatine for 30 min, followed by washing with PB (pH 7.5) two times for 2 min and incubation with 0.15% glycine for 2 min. The grids were incubated in blocking solution-I (1% BSA in PB) to avoid non-specific antigen binding. After three washing steps with wash buffer containing 0.1% acetylated BSA (BSA-c) in PB, the samples were incubated with anti-GRP78 antibody (#ab21685; Abcam) in blocking solution-II (0.15% BSA-c in PB) for 1 h, followed by five times washing in blocking solution-II for 2 min. Thereafter, the samples were incubated with protein A-gold 10 nm for 30 min, washed in PB five times for 2 min, fixed in 1% GA for 5 min, and washed in MilliQ 6 times for 1 min. Finally, the grids were stained on drops of 0.4% Uranyl acetate and 1.8% methylcellulose for 8 min, picked up, and blotted. The grids were analyzed in a JEM1400-LaB6 Transmission Electron Microscope (JEOL; Tokyo, Japan) operated at 80 kV and imaged with a EMSIS Quemesa 11 Mpxl camera.

### Co-immunoprecipitation assay

In order to identify the different interacting partners for sGRP78, control and cytokine-exposed INS-1E cells were subjected to a membrane fractionation isolation as described above. Subsequently, membrane proteins were eluted in 2 mM D-biotin (in PBS, ThermoFisher scientific) by centrifugation at 2000 rpm for 4 min at 4 °C. The eluate was then incubated with 6 µg of anti-GRP78 antibody (#sc-1050, Santa Cruz Biotechnology) overnight at 4 °C, followed by incubation with 30 µl A/G PLUS-agarose beads (#sc-2003, Santa Cruz Biotechnology) for 4 h at 4 °C. The beads were washed three times with pre-urea buffer (50 mM Tris; pH 8.5, 1 mM EGTA, and 75 mM KCl), followed by elution in urea buffer (7 M urea, 20 mM Tris; pH 7.5 and 100 mM NaCl). The proteins obtained were identified by LC-MS/MS on an Orbitrap Q Exactive (ThermoFisher scientific) as previously described^3^.

### siRNA silencing

*Dnajc3* was knocked down in MIN6 cells using ON-TARGETplus SMARTpool *Dnajc3* siRNA (#L-167232-00-0005; Dharmacon, Cambridge, UK). MIN6 cells were reverse transfected with 10 nM *Dnajc3* siRNA SMARTpool and 10 nM Non-targeting Control Pool (#D-001810-10, Dharmacon) using RNAiMAX lipofectamine (#13778150; Invitrogen) as per the manufacturer's instructions. Briefly, the cells were trypsinized, washed and suspended in DMEM and incubated at 37 °C. At the same time, the solution of siRNA, lipofectamine, and OptiMEM (#31985070; ThermoFisher scientific) was prepared and incubated for 15 min at RT. The above solution was mixed with 0.5 × 10^6^ cells in culture medium and the cells were cultured in 6-well plates for 48 h. Percentage of silencing was evaluated by quantitative reverse transcription PCR (qRT-PCR) (as described below).

### sGRP78 blocking

INS-1E cells were exposed to anti-GRP78 antibodies against the N-terminal domain (mouse anti-GRP78; #sc-376768; Santa Cruz Biotechnology), C-terminal domain (rabbit anti-GRP78; #ADI-SPA-826-F; Enzo life sciences, Antwerp, Belgium)), control polyclonal rabbit IgG (#BE0095, Bioxcell, NH, USA), and/or recombinant GRP78 (# ADI-SPP-765; Enzo life sciences, Antwerp, Belgium) in the presence of cytokines for 16 h. Prior to use, recombinant GRP78 was desalted to remove dithiothreitol and glycerol using Zeba spin columns with a molecular weight cutoff of 40 kDa (ThermoFisher Scientific) and reconstituted in 33 mM Tris-HCl, pH 7.5, and 33 mM NaCl. After incubation, total RNA was isolated and analyzed for stress markers with qRT-PCR (as described below). In parallel, cells were grown in 96-well plates for Hoechst-PI staining and Caspase-Glo® 3/7 Assay.

### Real-time qRT-PCR

Total RNA from cell lines or primary islets was isolated after the indicated treatments by using the High Pure RNA Isolation Kit (# 11828665001, Roche Diagnostics, Risch-Rotkreuz, Switzerland) and cDNA was prepared from 500 ng RNA (for cells) or 150 ng RNA (for islets) using oligo-d(T) and superscript II reverse transcriptase (# 18064014, Invitrogen). qRT-PCR was performed in a total volume of 10 µl, containing 4 pmol of forward and reverse primers, 5 µl Fast SYBR® Green Master Mix (# 4385612, Applied Biosystems, CA, USA), and 0.2 μl cDNA. The samples were assayed on an ABI-prism 7500 Fast (Applied Biosystems). The relative expression level of the genes of interest was normalized to the geometrical mean of three housekeeping genes, namely, hypoxanthine-guanine phosphoribosyltransferase (Hprt), 60S ribosomal protein L27 (RPL27), and beta-actin. The ∆∆Ct method was used for quantification. The amplification efficiency was equal for all genes. The primer sequences are provided in Supplementary Table S2.

### Statistics

Statistical analyses were performed using GraphPad Prism 7 (GraphPad Software, San Diego, CA). The results are presented as mean ± standard error of the mean (SEM) of independent experiments (*n*). Data were analyzed by two-tailed unpaired Student’s *t* test for comparison between two groups or one-way analysis of variance (ANOVA) followed by Sidak posthoc test for multiple group comparisons. Time-course experiments were analyzed by two-way ANOVA followed by Sidak posthoc test for multiple group comparisons (factors: time (4, 8, 16, and 24 h) and group (control, cytokine-exposed)). *P* values <0.05 were considered significant. Selection of sample size was based on our experience with previous and similar in vitro experiments and no samples were excluded from the analyses.

## Supplementary information

Figure S1

Figure S2

Figure S3

Figure S4

Figure S5

Supplementary Table S1

Supplementary Table S2

Supplementary Table S3

Supplementary figure legends
