# Supplementary material for: Cytokine-induced translocation of GRP78 to the plasma membrane triggers a pro-apoptotic feedback loop in pancreatic beta cells
Source: Cell Death Dis. 2019 Apr 5;10(4):309. doi: 10.1038/s41419-019-1518-0 (PMC6450900; doi:10.1038/s41419-019-1518-0)
Supplement: Supplementary file 6 — Supplementary Table S1 [file 41419_2019_1518_MOESM6_ESM.docx]

**Supplementary Table S1:** Characteristics of islet donors.

| Donor | Gender  (M/F) | Age  (years) | BMI  (kg/m^2^) | HbA1c  (% (mmol/mol)) | Cold ischemia time  (hours) |
| --- | --- | --- | --- | --- | --- |
| D1 | M | 53 | 34.1 | 5.6 (38) | 7.9 |
| D2 | M | 23 | 21.9 | 5.9 (41) | 20.2 |
| D3 | F | 62 | 28.3 | 6.1 (43) | 13.8 |
| D4 | F | 60 | 36.4 | 6.2 (44) | 18.0 |
| D5 | F | 49 | 22.0 | 5.6 (38) | 0.9 |
