# Supplementary material for: Cytokine-induced translocation of GRP78 to the plasma membrane triggers a pro-apoptotic feedback loop in pancreatic beta cells
Source: Cell Death Dis. 2019 Apr 5;10(4):309. doi: 10.1038/s41419-019-1518-0 (PMC6450900; doi:10.1038/s41419-019-1518-0)
Supplement: Supplementary file 7 — Supplementary Table S2 [file 41419_2019_1518_MOESM7_ESM.docx]

**Supplementary Table S2:** Primers used for qRT-PCR

|  | **Species** | **Gene** | **Primer** | **Sequence (5'-3')** |
| --- | --- | --- | --- | --- |
| 1 | Human | Beta-ACTIN | FP | ACCCCAAGGCCAACCG |
| 2 |  |  | RP | ACAGCCTGGATAGCAACGTACA |
| 3 |  | HPRT | FP | TGTAGGATATGCCCTTGACTATA |
| 4 |  |  | RP | CAATAGGACTCCAGATGTTTCCA |
| 5 |  | RPL27 | FP | TGTCGTCAATAAGGATGTCTTCAGA |
| 6 |  |  | RP | TGCCTGTCTTGTATCTCTCTTCAAA |
| 7 |  | CHOP | FP | GAACGGCTCAAGCAGGAAATC |
| 8 |  |  | RP | TTCACCATTCGGTCAATCAGAG |
| 9 | Mouse | Beta-Actin | FP | AGAGGGAAATCGTGCGTGAC |
| 10 |  |  | RP | CAATAGTGATGACCTGGCCGT |
| 11 |  | Hprt | FP | TGGCCATCTGCCTAGTAAAGC |
| 12 |  |  | RP | GGCTCATAGTGCAAATCAAAAGTC |
| 13 |  | Rpl27 | FP | GTCGAGATGGGCAAGTTCAT |
| 14 |  |  | RP | TTCTTCACGATGACGGCTTT |
| 15 |  | Chop | FP | TCTCATCCCCAGGAAACGAA |
| 16 |  |  | RP | ATCTGGAGAGCGAGGGCTTT |
| 17 | Rat | Dnajc3 | FP | GCCGATGCCTTATCTCAGTT |
| 18 |  |  | RP | GCAGTGCTGCTTTGGATTTT |
| 19 |  | Hprt | FP | GTCAACGGGGGACATAAAAG |
| 20 |  |  | RP | GCTTGACCAAGGAAAGCAAA |
| 21 |  | Rpl27 | FP | CCTCATGCCCACAAGGTACTC |
| 22 |  |  | RP | TCTTGTATCGCTCCTCAAACTTGA |
| 23 |  | Chop | FP | CCAGCAGAGGTCACAAGCAC |
| 24 |  |  | RP | CGCACTGACCACTCTGTTTC |
| 25 |  | Dp5 | FP | GGCACCCAAATGGCAAACTA |
| 26 |  |  | RP | CCTTCACCCATAATGCTTGCTT |
| 27 |  | Atf3 | FP | TCTCACCGGCTCCAGGATT |
| 28 |  |  | RP | ATTAGTGCGATCCTGCTTTGC |
| 29 |  | Bax | FP | GGGAGCGGCTGCTTGTC |
| 30 |  |  | RP | AGCCACAAAGATGGTCACTGTCT |
| 31 |  | Mcl1 | FP | TGAAAAGGGTGAATGGAAAGGT |
| 32 |  |  | RP | CCATTGTTAGGGCTTCTCTGTCA |
|  |  | Ins2 | FP | GCTGGCCCTGCTCATCCT |
|  |  |  | RP | CCACCAAGTGAGAACCACAAAG |
