# Supplementary material for: Cytokine-induced translocation of GRP78 to the plasma membrane triggers a pro-apoptotic feedback loop in pancreatic beta cells
Source: Cell Death Dis. 2019 Apr 5;10(4):309. doi: 10.1038/s41419-019-1518-0 (PMC6450900; doi:10.1038/s41419-019-1518-0)
Supplement: Supplementary file 8 — Supplementary Table S3 [file 41419_2019_1518_MOESM8_ESM.docx]

**Supplementary Table S3**: Identified sGRP78-interacting proteins in Control and Cytokines exposed INS-1E cells.

| **Control** | | |
| --- | --- | --- |
| **Protein Names** | **SwissProt ID** | **Gene Names** |
| Actin, cytoplasmic 1 | Q71FK5 | ACTB |
| ADP/ATP translocase 1 | P48962 | Slc25a4 |
| ADP/ATP translocase 2 | P51881 | Slc25a5 |
| Alpha-fetoprotein | P02773 | Afp |
| Annexin A2 | Q07936 | Anxa2 |
| Arf-GAP with SH3 domain | Q7SIG6 | Asap2 |
| ATP synthase subunit alpha | P15999 | Atp5f1a |
| ATP-dependent RNA helicase DDX1 | Q641Y8 | Ddx1 |
| Calmodulin-1 | P0DP26 | Calm1 |
| Calumenin | Q4U471 | CALU |
| Calumenin | O35783 | Calu |
| cAMP-dependent protein kinase type I-alpha regulatory subunit | P86244 | PRKAR1A |
| Collagen alpha-1 | P02454 | Col1a1 |
| Complement C4 | P08649 | C4 |
| Cytochrome P450 1A2 | Q64391 | CYP1A2 |
| DAZ-associated protein 1 | Q9JII5 | Dazap1 |
| Desmoplakin | E9Q557 | Dsp |
| Elongation factor Tu, mitochondrial | P85834 | Tufm |
| Endoplasmic reticulum chaperone BiP | P07823 | HSPA5 |
| Erlin-2 | Q8BFZ9 | Erlin2 |
| Glucagon | P55095 | Gcg |
| Heat shock cognate 71 kDa protein | P19378 | HSPA8 |
| Hemoglobin subunit alpha | P20854 | HBA |
| Hemoglobin subunit beta | P02095 | HBB |
| Heterogeneous nuclear ribonucleoprotein A/B | Q99020 | Hnrnpab |
| Heterogeneous nuclear ribonucleoprotein A0 | Q9CX86 | Hnrnpa0 |
| Heterogeneous nuclear ribonucleoprotein A1 | P49312 | Hnrnpa1 |
| Heterogeneous nuclear ribonucleoprotein D-like | Q9Z130 | Hnrnpdl |
| Heterogeneous nuclear ribonucleoprotein F | Q9Z2X1 | Hnrnpf |
| Heterogeneous nuclear ribonucleoprotein H | O35737 | Hnrnph1 |
| Heterogeneous nuclear ribonucleoprotein U | Q8VEK3 | Hnrnpu |
| High mobility group protein B4 | Q6P8W9 | Hmgb4 |
| Histone H1.1 | D4A3K5 | Hist1h1a |
| Histone H1.2 | P15864 | Hist1h1c |
| Histone H2B type 1-B | Q64475 | Hist1h2bb |
| Histone-binding protein RBBP4 | Q60972 | Rbbp4 |
| Histone-binding protein RBBP7 | Q60973 | Rbbp7 |
| Homogentisate 1,2-dioxygenase | O09173 | Hgd |
| Ig heavy chain Mem5 | P84751 | Ighg2a |
| Insulin-1 | P01322 | Ins1 |
| Junction plakoglobin | Q02257 | Jup |
| Large neutral amino acids transporter small subunit 1 | Q63016 | Slc7a5 |
| Leukocyte surface antigen CD47 | P97829 | Cd47 |
| Neuroendocrine convertase 2 | P21661 | Pcsk2 |
| Neutral alpha-glucosidase AB | Q8BHN3 | Ganab |
| Nuclease-sensitive element-binding protein 1 | P62960 | Ybx1 |
| Nucleobindin-1 | Q02819 | Nucb1 |
| Nucleobindin-2 | Q9JI85 | Nucb2 |
| Nucleolin | P13383 | Ncl |
| Nucleophosmin | Q61937 | Npm1 |
| Peroxiredoxin-2 | Q8K3U7 | PRDX2 |
| Peroxisome proliferator-activated receptor delta | P35396 | Ppard |
| Polyadenylate-binding protein 1 | P29341 | Pabpc1 |
| Probable ATP-dependent RNA helicase DDX5 | Q61656 | Ddx5 |
| ProSAAS | Q9QXV0 | Pcsk1n |
| Pyruvate carboxylase, mitochondrial | P52873 | Pc |
| Reticulocalbin-2 | Q8BP92 | Rcn2 |
| Secretogranin-1 | O35314 | Chgb |
| Serine/arginine-rich splicing factor 1 | Q6PDM2 | Srsf1 |
| Serine/arginine-rich splicing factor 2 | Q62093 | Srsf2 |
| Serine/arginine-rich splicing factor 5 | O35326 | Srsf5 |
| Serum albumin | P07724 | Alb |
| Sodium/potassium-transporting ATPase subunit alpha-1 | Q8VDN2 | Atp1a1 |
| Sodium/potassium-transporting ATPase subunit alpha-2 | Q6PIE5 | Atp1a2 |
| Sodium/potassium-transporting ATPase subunit beta-1 | P14094 | Atp1b1 |
| Solute carrier family 2, facilitated glucose transporter member 2 | P12336 | Slc2a2 |
| Sphingosine-1-phosphate lyase 1 | Q8CHN6 | Sgpl1 |
| Synaptotagmin-like protein 4 | Q8VHQ7 | Sytl4 |
| TAR DNA-binding protein 43 | Q921F2 | Tardbp |
| Thioredoxin domain-containing protein 12 | Q9CQU0 | Txndc12 |
| Trifunctional enzyme subunit alpha, mitochondrial | Q64428 | Hadha |
| Trifunctional enzyme subunit beta, mitochondrial | Q60587 | Hadhb |
| Tubulin alpha-1A chain | P68362 | TUBA1A |
| Tubulin beta-4B chain | P68372 | Tubb4b |
| Tubulin beta-5 chain | P69893 | TUBB5 |
| Ubiquitin-40S ribosomal protein S27a | P62978 | RPS27A |
| Ubiquitin-associated protein 2-like | Q80X50 | Ubap2l |
| 40S ribosomal protein S13 | P62301 | Rps13 |
| 40S ribosomal protein S14 | P62265 | RPS14 |
| 40S ribosomal protein S15a | P62245 | Rps15a |
| 40S ribosomal protein S16 | P14131 | Rps16 |
| 40S ribosomal protein S17 | P63274 | RPS17 |
| 40S ribosomal protein S18 | P62270 | Rps18 |
| 40S ribosomal protein S2 | P25444 | Rps2 |
| 40S ribosomal protein S24 | P62848 | RPS24 |
| 40S ribosomal protein S26 | P30742 | RPS26 |
| 40S ribosomal protein S28 | P62858 | Rps28 |
| 40S ribosomal protein S3 | P62908 | Rps3 |
| 40S ribosomal protein S4, X isoform | P62704 | RPS4X |
| 40S ribosomal protein S6 | P62754 | Rps6 |
| 40S ribosomal protein S8 | P62242 | Rps8 |
| 40S ribosomal protein S9 | Q6ZWN5 | Rps9 |
| 4F2 cell-surface antigen heavy chain | Q794F9 | Slc3a2 |
| 60S acidic ribosomal protein P0 | P19945 | Rplp0 |
| 60S acidic ribosomal protein P1 | P19944 | Rplp1 |
| 60S acidic ribosomal protein P2 | P02401 | Rplp2 |
| 60S ribosomal protein L10a | P53026 | Rpl10a |
| 60S ribosomal protein L11 | Q6QMZ8 | RPL11 |
| 60S ribosomal protein L12 | Q6QMZ7 | RPL12 |
| 60S ribosomal protein L13 | P47963 | Rpl13 |
| 60S ribosomal protein L14 | Q63507 | Rpl14 |
| 60S ribosomal protein L15 | Q9CZM2 | Rpl15 |
| 60S ribosomal protein L18 | P12001 | Rpl18 |
| 60S ribosomal protein L19 | P84099 | Rpl19 |
| 60S ribosomal protein L24 | Q8BP67 | Rpl24 |
| 60S ribosomal protein L27 | P61358 | Rpl27 |
| 60S ribosomal protein L29 | P47915 | Rpl29 |
| 60S ribosomal protein L3 | P21531 | Rpl3 |
| 60S ribosomal protein L31 | Q1KSC7 | RPL31 |
| 60S ribosomal protein L34 | Q9D1R9 | Rpl34 |
| 60S ribosomal protein L35 | Q6ZWV7 | Rpl35 |
| 60S ribosomal protein L35a | O55142 | Rpl35a |
| 60S ribosomal protein L4 | Q9D8E6 | Rpl4 |
| 60S ribosomal protein L4 | P50878 | Rpl4 |
| 60S ribosomal protein L6 | P21533 | Rpl6 |
| 60S ribosomal protein L7 | P14148 | Rpl7 |
| 60S ribosomal protein L7a | P12970 | Rpl7a |
| 60S ribosomal protein L8 | P62918 | Rpl8 |
| 60S ribosomal protein L9 | P51410 | Rpl9 |

| **Cytokine** | | |
| --- | --- | --- |
| **Protein Names** | **SwissProt ID** | **Gene Names** |
| ADP/ATP translocase 1 | P48962 | Slc25a4 |
| ADP/ATP translocase 2 | P51881 | Slc25a5 |
| ADP/ATP translocase 4 | Q3V132 | Slc25a31 |
| Alpha-2-macroglobulin-P | Q6GQT1 | A2m |
| Alpha-fetoprotein | P02773 | Afp |
| Annexin A2 | Q07936 | Anxa2 |
| Apolipoprotein A-I | Q00623 | Apoa1 |
| Arf-GAP with SH3 domain | Q7SIG6 | Asap2 |
| ATP synthase subunit alpha | P15999 | Atp5f1a |
| ATP synthase subunit beta | P10719 | Atp5f1b |
| ATP synthase subunit d | Q9DCX2 | Atp5h |
| ATP synthase subunit delta | Q9D3D9 | Atp5f1d |
| ATP synthase-coupling factor 6 | P21571 | Atp5j |
| Calmodulin-1 | P0DP26 | Calm1 |
| Calumenin | Q4U471 | CALU |
| Carboxypeptidase E | Q00493 | Cpe |
| Class I histocompatibility antigen | P15978 | RT1-Aw2 |
| Collagen alpha-1 | P02454 | Col1a1 |
| Complement C3 | P01027 | C3 |
| Complement C4 | P08649 | C4 |
| Cytochrome c oxidase subunit 5A | P12787 | Cox5a |
| Cytochrome c oxidase subunit 5B | P12075 | Cox5b |
| Cytochrome P450 1A2 | Q64391 | CYP1A2 |
| DAZ-associated protein 1 | Q9JII5 | Dazap1 |
| Desmoplakin | E9Q557 | Dsp |
| **DnaJ homolog subfamily C member 3** | **Q91YW3** | **Dnajc3** |
| Elongation factor 1-beta | O70251 | Eef1b |
| Elongation factor 1-delta | P57776 | Eef1d |
| Elongation factor 1-gamma | Q68FR6 | Eef1g |
| Elongation factor Tu | P85834 | Tufm |
| Endoplasmic reticulum chaperone BiP | P20029 | Hspa5 |
| Erlin-2 | Q8BFZ9 | Erlin2 |
| Gem-associated protein 5 | Q8BX17 | Gemin5 |
| Glucagon | P05110 | GCG |
| Glutaminase kidney isoform | D3Z7P3 | Gls |
| Golgin subfamily A member 2 | Q62839 | Golga2 |
| Heat shock cognate 71 kDa protein | P19378 | HSPA8 |
| Heat shock protein HSP 90-beta | P11499 | Hsp90ab1 |
| Hemoglobin subunit alpha | P20854 | HBA |
| Hemoglobin subunit beta | P02095 | HBB |
| Heterogeneous nuclear ribonucleoprotein A/B | Q99020 | Hnrnpab |
| Heterogeneous nuclear ribonucleoprotein A0 | Q9CX86 | Hnrnpa0 |
| Heterogeneous nuclear ribonucleoprotein A1 | P49312 | Hnrnpa1 |
| Heterogeneous nuclear ribonucleoprotein A3 | Q8BG05 | Hnrnpa3 |
| Heterogeneous nuclear ribonucleoprotein D-like | Q9Z130 | Hnrnpdl |
| Heterogeneous nuclear ribonucleoprotein F | Q9Z2X1 | Hnrnpf |
| Heterogeneous nuclear ribonucleoprotein H | O35737 | Hnrnph1 |
| Heterogeneous nuclear ribonucleoprotein H2 | P70333 | Hnrnph2 |
| Heterogeneous nuclear ribonucleoprotein K | P61979 | Hnrnpk |
| Heterogeneous nuclear ribonucleoprotein M | Q62826 | Hnrnpm |
| Heterogeneous nuclear ribonucleoprotein Q | Q7TMK9 | Syncrip |
| Heterogeneous nuclear ribonucleoprotein U | Q8VEK3 | Hnrnpu |
| Histone H1.1 | D4A3K5 | Hist1h1a |
| Ig gamma-1 chain C region | P01869 | Ighg1 |
| Ig heavy chain Mem5 | P84751 | Ighg2a |
| Intercellular adhesion molecule 1 | Q00238 | Icam1 |
| Leukocyte surface antigen CD47 | P97829 | Cd47 |
| Malate dehydrogenase | P08249 | Mdh2 |
| MICOS complex subunit Mic60 | Q3KR86 | Immt |
| NADH dehydrogenase | P19234 | Ndufv2 |
| Neuroendocrine convertase 2 | P21661 | Pcsk2 |
| Nuclease-sensitive element-binding protein 1 | P62960 | Ybx1 |
| Nucleobindin-2 | Q9JI85 | Nucb2 |
| Nucleolin | P13383 | Ncl |
| Nucleophosmin | Q61937 | Npm1 |
| Peroxiredoxin-1 | Q9JKY1 | PRDX1 |
| Peroxiredoxin-2 | Q8K3U7 | PRDX2 |
| Peroxiredoxin-4 | O08807 | Prdx4 |
| Peroxisome proliferator-activated receptor delta | P35396 | Ppard |
| Plasminogen activator inhibitor 1 RNA-binding protein | Q9CY58 | Serbp1 |
| Polyadenylate-binding protein 1 | P29341 | Pabpc1 |
| Polypyrimidine tract-binding protein 1 | P17225 | Ptbp1 |
| Probable ATP-dependent RNA helicase DDX17 | Q501J6 | Ddx17 |
| Probable ATP-dependent RNA helicase DDX5 | Q61656 | Ddx5 |
| Prohibitin | P86220 | PHB |
| Protein disulfide-isomerase | P09103 | P4hb |
| Protein disulfide-isomerase A3 | P86235 | PDIA3 |
| Protein disulfide-isomerase A6 | Q922R8 | Pdia6 |
| Protein TANC2 | A2A690 | Tanc2 |
| Pyruvate carboxylase | Q05920 | Pc |
| Reticulocalbin-2 | Q8BP92 | Rcn2 |
| RT1 class I histocompatibility antigen | P16391 | RT1-EC2 |
| Sarcoplasmic/endoplasmic reticulum calcium ATPase 2 | O55143 | Atp2a2 |
| Serine/arginine-rich splicing factor 1 | Q6PDM2 | Srsf1 |
| Serine/arginine-rich splicing factor 3 | P84104 | Srsf3 |
| Serum albumin | P07724 | Alb |
| Sodium/potassium-transporting ATPase subunit alpha-1 | Q8VDN2 | Atp1a1 |
| Sodium/potassium-transporting ATPase subunit beta-1 | P14094 | Atp1b1 |
| Solute carrier family 2, facilitated glucose transporter member 2 | P12336 | Slc2a2 |
| Sphingosine-1-phosphate lyase 1 | Q8CHN6 | Sgpl1 |
| Synaptotagmin-like protein 4 | Q8VHQ7 | Sytl4 |
| TAR DNA-binding protein 43 | Q921F2 | Tardbp |
| Thioredoxin domain-containing protein 12 | Q9CQU0 | Txndc12 |
| Transcriptional activator protein Pur-beta | Q68A21 | Purb |
| Transferrin receptor protein 1 | Q62351 | Tfrc |
| Translocon-associated protein subunit alpha | Q9CY50 | Ssr1 |
| Trifunctional enzyme subunit alpha | Q64428 | Hadha |
| Trifunctional enzyme subunit beta | Q99JY0 | Hadhb |
| Tubulin alpha-1A chain | P68362 | TUBA1A |
| Tubulin beta-4B chain | P68372 | Tubb4b |
| Tubulin beta-5 chain | P69893 | TUBB5 |
| Ubiquitin-associated protein 2-like | Q80X50 | Ubap2l |
| Voltage-dependent anion-selective channel protein 1 | Q60932 | Vdac1 |
| Voltage-dependent anion-selective channel protein 2 | Q60930 | Vdac2 |
| Voltage-dependent anion-selective channel protein 3 | Q60931 | Vdac3 |
| 40S ribosomal protein S10 | P63325 | Rps10 |
| 40S ribosomal protein S13 | P62301 | Rps13 |
| 40S ribosomal protein S14 | P62265 | RPS14 |
| 40S ribosomal protein S15a | P62245 | Rps15a |
| 40S ribosomal protein S16 | P14131 | Rps16 |
| 40S ribosomal protein S17 | P63274 | RPS17 |
| 40S ribosomal protein S18 | P62270 | Rps18 |
| 40S ribosomal protein S2 | P25444 | Rps2 |
| 40S ribosomal protein S24 | P62848 | RPS24 |
| 40S ribosomal protein S26 | P30742 | RPS26 |
| 40S ribosomal protein S28 | P62858 | Rps28 |
| 40S ribosomal protein S3 | P62908 | Rps3 |
| 40S ribosomal protein S3a | P97351 | Rps3a |
| 40S ribosomal protein S4, X isoform | P62704 | RPS4X |
| 40S ribosomal protein S5 | P97461 | Rps5 |
| 40S ribosomal protein S6 | P62754 | Rps6 |
| 40S ribosomal protein S7 | P62082 | Rps7 |
| 40S ribosomal protein S8 | P62242 | Rps8 |
| 4F2 cell-surface antigen heavy chain | Q794F9 | Slc3a2 |
| 60 kDa heat shock protein | P18687 | HSPD1 |
| 60S acidic ribosomal protein P0 | P19945 | Rplp0 |
| 60S acidic ribosomal protein P1 | P19944 | Rplp1 |
| 60S acidic ribosomal protein P2 | P02401 | Rplp2 |
| 60S ribosomal protein L10a | P53026 | Rpl10a |
| 60S ribosomal protein L11 | Q6QMZ8 | RPL11 |
| 60S ribosomal protein L12 | Q6QMZ7 | RPL12 |
| 60S ribosomal protein L13 | P47963 | Rpl13 |
| 60S ribosomal protein L14 | Q63507 | Rpl14 |
| 60S ribosomal protein L15 | Q9CZM2 | Rpl15 |
| 60S ribosomal protein L18 | P12001 | Rpl18 |
| 60S ribosomal protein L24 | Q8BP67 | Rpl24 |
| 60S ribosomal protein L26 | P61255 | Rpl26 |
| 60S ribosomal protein L27 | P61358 | Rpl27 |
| 60S ribosomal protein L29 | P25886 | Rpl29 |
| 60S ribosomal protein L3 | P27659 | Rpl3 |
| 60S ribosomal protein L31 | Q1KSC7 | RPL31 |
| 60S ribosomal protein L35 | Q6ZWV7 | Rpl35 |
| 60S ribosomal protein L35a | O55142 | Rpl35a |
| 60S ribosomal protein L4 | Q9D8E6 | Rpl4 |
| 60S ribosomal protein L4 | P50878 | Rpl4 |
| 60S ribosomal protein L6 | P21533 | Rpl6 |
| 60S ribosomal protein L7 | P14148 | Rpl7 |
| 60S ribosomal protein L7a | P12970 | Rpl7a |
| 60S ribosomal protein L8 | P62918 | Rpl8 |
