# Supplementary material for: Cytokine-induced translocation of GRP78 to the plasma membrane triggers a pro-apoptotic feedback loop in pancreatic beta cells
Source: Cell Death Dis. 2019 Apr 5;10(4):309. doi: 10.1038/s41419-019-1518-0 (PMC6450900; doi:10.1038/s41419-019-1518-0)
Supplement: Supplementary file 9 — Supplementary figure legends [file 41419_2019_1518_MOESM9_ESM.docx]

**Supplementary figure legends**

**Fig S1: Flow cytometry gating strategy and population plots.**

**a**: FACS gating strategy for the identification of living, early apoptotic and late apoptotic cells. First, MIN6 cells were gated using FSC-H and SSC-H and then gated for single cell population using FSC-H vs. FSC-W and SSC-H vs. SSC-W scatter plots. Living, early apoptotic and late apoptotic cells were then gated based on Annexin-V and DRAQ7 expression, with Annexin-V^-^ DRAQ7^-^ defined as the living cell population, Annexin-V^+^ DRAQ7^-^ defined as the early apoptotic cell population and Annexin-V^+^ DRAQ7^+^ defined as late apoptotic cells. Subsequently, each population was gated for GRP78 positive cells. **b:** Contour plots showing shifting of living cell~~s~~ population to early apoptotic cell population upon exposure to cytokines (Cyt) during 4h, 8h, 16h and 24h in MIN6 cells.

**Fig S2: Surface GRP78 Interacting proteins identification through LC-MS/MS of surface proteins.**

**a:** The strategy for the identification of surface GRP78 interacting proteins through LC-MS/MS. **b:** The Venn diagram for the number of proteins identified with LC-MS/MS from control and cytokine-exposed (16 h) INS-1E cells as surface GRP78 interacting proteins. **c:** Relative *Dnajc3* mRNA expression in MIN6 cells transfected with *Dnajc3* siRNA for 48 h, as compared to control MIN6 cells transfected with scrambled siRNA. Data are presented as mean ± SEM (n=3) and analyzed by two-tailed unpaired Student’s *t*-test. Significance is indicated by ***P<0.001.

**Fig S3: Soluble GRP78 dose-dependently increases cell death in beta cells.**

Apoptosis levels in INS-1E cells upon exposure to cytokines (hIL-1β (50 U/ml), rIFN-γ (500 U/ml)) and recombinant GRP78 with indicated doses (16 h; n=10). Data are presented as mean ± SEM and statistically analyzed by a one-way ANOVA followed by Sidak postHoc test for multiple group comparisons. Significance is indicated by *P<0.05.

**Fig S4: Blocking of sGRP78 partially protects beta cell dysfunction.**

Relative mRNA expression of *Ins2* in cytokine-exposed INS-1E cells (hIL-1β (50 U/ml), rIFN-γ (500 U/ml)) with or without anti-GRP78 blocking antibodies against the N-terminal region (N-term, 5 μg/ml) (n=4), or C-terminal region (C-term, 10 μg/ml) (n=5) for 16 h. Data are presented as mean ± SEM and statistically analyzed by a one-way ANOVA followed by Sidak postHoc test for multiple group comparisons. Significance is indicated by *P<0.05, **P<0.01, ~~***P<0.001~~ and ****P<0.0001.

**Fig S5: Soluble GRP78 increases pro-apoptotic stress markers.**

Relative mRNA expression of *Dp5* **(a)**, *Atf3* **(b)** and *Mcl1* **(c)** in cytokine-exposed INS-1E cells (hIL-1β (50 U/ml), rIFN-γ (500 U/ml) in the presence of recombinant GRP78 (1μM) with or without anti-GRP78 blocking antibodies against the N-terminal region (N-term, 5 μg/ml), or C-terminal region (C-term, 10 μg/ml) for 16 h (n=4). Data are presented as mean ± SEM and statistically analyzed by a one-way ANOVA followed by Sidak postHoc test for multiple group comparisons. Significance is indicated by *P<0.05, **P<0.01, ***P<0.001 and ****P<0.0001.

**Supplementary Table S1: Characteristics of islet donors.**

**Supplementary Table S2: Primers used for qRT-PCR.**

**Supplementary Table S3: Identified sGRP78-interacting proteins in control and cytokine-exposed INS-1E cells**

Potential GRP78-interacting proteins were identified by LC-MS/MS analysis of immunoprecipitates from membrane fractions of control and cytokine-exposed INS-1E cells. Protein Names, SwissProt ID and Gene Names are according to UniProt (http://www.uniprot.org).
